# From Grief to Growth: The Role of Coping Strategies, Kinship and Cause of Death

**DOI:** 10.1177/00302228241259647

**Published:** 2024-06-06

**Authors:** Marta Pereira, Ana Moreira, David Dias Neto

**Affiliations:** 1 ISPA - Instituto Universitário, Lisboa, Portugal; 2 Faculdade de Ciências e Tecnologia, 126808 Universidade Europeia, Lisboa, Portugal; 3 APPsyCI - Applied Psychology Research Center Capabilities & Inclusion, Lisboa, Portugal

**Keywords:** grief, post-traumatic growth, coping strategies

## Abstract

**Background:** Research has predominantly focused on the post-traumatic consequences of grief. Less is known about the factors associated with the capacity for recovery and growth. **Objective:** The main goal of this study is to analyse the mediating role of coping strategies in the relationship between the impact of the event and posttraumatic growth, considering the degree of kinship and the cause of death. **Methods:** This exploratory and correlational study, which used a cross-sectional design, involved a community sample of 889 adults who had lost a loved one. **Results:** Emotion and problem-focused coping strategies mediate the relationship between the impact of loss and posttraumatic growth, specifically when a spouse or a child dies. **Conclusions:** Individuals who experience greater suffering also undergo higher levels of post-traumatic growth. Coping strategies are crucial in post-trauma reconstruction and growth. Furthermore, the degree of kinship and the cause of death are decisive factors.

## Introduction

Posttraumatic growth (PTG) has been gaining prominence in the study of grief. While a growing body of research over the past decade indicates a strong association between these concepts, there remains a significant gap in our understanding of PTG, specifically in the context of grief following the loss of a loved one (Michael & Cooper, 2013). While there is evidence about the various ways people may respond during the grieving process, it is crucial to also take into account the circumstances and type of death, as these factors may serve as either risk or protective factors influencing the development of different grief patterns (Carqueja, 2017).

The concept of PTG refers to a positive transformation resulting from facing a traumatic life event (Calhoun et al., 2000). This construct involves changes in beliefs, goals, behaviours and identity, encompassing cognitive processes like reconstructing schemas and seeking meaning (Tedeschi & Calhoun, 1996, 2004b). Growth depends on individuals’ subjective interpretation after their traumatic experience and the degree of severity attributed to the event (Morris et al., 2005). For the interpretation of the event and the consequent experience of PTG to exist, the presence of pre-existing schemas before the trauma and their subsequent alteration is necessary (Tedeschi & Calhoun, 2004a).

Grief requires a reconstruction of meanings challenged by loss, as described by Tedeschi and Calhoun in the PTG model of grief (Calhoun et al., 2010). When individuals face impactful events that challenge their beliefs, they experience distress and re-evaluate their core principles The significant disruptions in core beliefs are linked to a higher degree of growth. So, even though distress levels and PTSD are higher, there is a greater chance of experiencing PTG (Calhoun et al., 2010). Furthermore, in the process of this reconstruction, additional factors can influence the experience of PTG. Empirical findings indicate the presence of mediating factors that are associated with an increased likelihood of PTG emerging when individuals confront the loss of a loved one (Michael & Cooper, 2013).

Among these mediator factors, it is important to highlight coping strategies. For example, a literature review showed that rumination and intrusions were positively associated with growth, highlighting that cognitive processing is essential for reconstructing shattered perspectives after trauma (Linley & Joseph, 2004). Specifically, active problem-solving (cognitive restructuring) enhances individuals’ ability to embrace new opportunities and, consequently, their growth following loss (Wolchik et al., 2008). So, consistent research has revealed that cognitive mechanisms such as active coping strategies, the creation of meaning and sense, and rumination can play a fundamental role in the experience of PTG (Michael & Cooper, 2013).

Some individuals tend to show maladaptive patterns, while others appear to have the capacity to perceive and experience positive changes (Tedeschi & Calhoun, 2004b). Coping is generally viewed as an intermediary variable mediating the impact of an event in its subsequent consequences or outcomes (Ribeiro & Rodrigues, 2004). So, this presupposes that specific coping strategies reduce the negative consequences of grief while others intensify them (Stroebe & Schut, 2001a). Predictions regarding the effectiveness of coping strategies in grief remain difficult to predict. Nevertheless, two types of general strategies hold particular relevance: problem-focused versus emotion-focused coping and confrontation versus avoidance (Stroebe & Schut, 2001b). Problem-focused coping requires an active role in managing and resolving the problem that causes distress, primarily focusing on finding a solution (Billings & Moos, 1981; Lazarus & Folkman, 1984). Emotion-focused coping regulates the emotional responses to the same event (Billings & Moos, 1981). An approach to emotion management can be executed by two distinct types of strategies: active and avoidant (Holahan & Moos, 1987). In this context, active emotional coping encompasses expressing emotions or the cognitive re-evaluation of the stressful situation. In contrast, avoidant emotional coping involves suppressing or evading emotional responses (Suar et al., 2017). Consequently, individuals who are grieving may avoid specific emotional reactions (e.g., crying over personal loss) while simultaneously confronting other facets of the experience (e.g., dealing with feelings of guilt) (Stroebe & Schut, 2001a). Coping strategies, due to their specificity, cannot be understood devoid of the context of their use.

Among grief reactions, two dimensions are particularly important: kinship and cause of death. The degree of kinship mediates how the bereaved individual perceives and is affected by the loss, affecting aspects such as well-being and health (Schaefer & Moos, 2001). A study showed that the loss of a child results in higher levels of depression and suffering compared to parental loss (Devries & Arbuckle, 2005) but could result in a reassessment of life and beneficial alterations in psychosocial well-being (Ogińska-Bulik & Kobylarczyk, 2019). However, in their research, Stroebe and Schut (2001b), losing a spouse is not typically associated with experiencing these positive changes. Losing a spouse involves many other losses, like shared life plans and support in raising children. In other words, and as we can see in another study, there appears to be a greater likelihood of experiencing high levels of prolonged grief when losing a spouse or a child (Thieleman et al., 2023). In another study, researchers found that when people lose one of their parents, it is more likely for more positive consequences to emerge, as the need to be independent fosters greater self-confidence and maturity (Leahy, 1997).

The cause of death is also important. Violent causes of death, such as suicide, homicide, and accidents, are linked to adverse grief outcomes (Sanders, 1988; Stroebe & Schut, 2001a; Thieleman et al., 2023). In line with these results, prior studies have shown that deaths perceived as traumatic, unexpected, or violent correlate with elevated levels of posttraumatic growth (Armstrong & Shakespeare-Finch, 2011; Currier et al., 2013; Yilmaz & Zara, 2016). In other words, the higher the perceived threat and damage levels, the greater the association with growth (Znoj, 2005). Since the variables mentioned above lack consistency, these underscores the significance of considering such factors in the eventual experience of PTG.

Understanding how the death of a significant other can bring about positive changes is important for personalising interventions (Lumb et al., 2017). The literature suggests that coping strategies are an important factor in determining whether death leads to growth or deterioration. But to understand the role of coping, it is essential to study the context in which these strategies are implemented. Considering the literature, two dimensions are important factors: kindship and cause of death. An integrated view exploring the mechanisms associated with growth is an important exploratory step in studying the phenomena of PTG after the death of a significant person.

Considering this, the objectives of the present study are as follows: 1. Analyse the different levels of PTG in sociodemographic and grief variables (gender, degree of kinship, and cause of death); 2. Evaluate the relationship between the impact of the loss of a loved one and PTG through the mediating effect of coping strategies, considering the cause of death and degree of kinship.

## Method

This study is a quantitative, descriptive, exploratory, and correlational study with a cross-sectional design.

### Participants

A sample comprising 889 adults (719 females and 170 males) was primarily recruited through social media platforms and websites. The sample employed in this research was derived from a non-probabilistic convenience sampling approach. The inclusion criteria stipulated the following prerequisites: having undergone the loss of a loved one, being 18 years old or above, and being proficient in reading and writing the Portuguese language.

The participants ‘ages ranged from 19 to 78 years (M = 43.74; SD = 13.26). Mainly were Portuguese (800, 97%), while the remaining individuals resided in Portugal and spoke Portuguese (27, 3%). Most participants were married or cohabiting (341, 38%), with a sizable portion being single (279, 31%). Additionally, a significant number had completed a degree (327,37%), while a substantial portion of the participants had finished their education after high school (220, 25%). Geographically, the participants were dispersed across all districts of Portugal, with Lisbon having the highest representation (511, 58%), followed by Aveiro. Since the goal was to analyze the mechanisms in a diverse set of contexts we aimed for a broad sample with respect to age, time of death and other dimensions.

### Measures

***Impact of event scale-Revised*** (IES-R; Brunet et al., 2003; Weiss & Marmar, 1997) was employed to assess the impact of a significant life event perceived as a major source of stress. Participants responded to the 22-item self-report questionnaire using a Likert scale, ranging from 0 (Not at all) to 4 (Extremely), indicating the frequency of experiencing the described symptoms in recent times. The IES-R provides total and subscale scores for Physiological Activation, Intrusion, Avoidance, and Emotional Anesthesia. The scale’s internal consistency was found to be satisfactory, with a Cronbach’s alpha coefficient of .92 for the total scale, indicating good reliability (Vieira et al., 2019). Higher scores on the scale indicate a greater level of subjective traumatic stress symptoms. In the present study, a similar level of internal consistency was observed, with a Cronbach’s Alpha value of .94 for the total scale.

***Brief-Cope*** is a condensed version of the original “COPE” inventory, designed to access coping styles and strategies (Carver, 1996). Ribeiro and Rodrigues (2004) adapted it for the Portuguese population. It consisted of 28 items categorised into 14 subscales, using a 4-point Likert scale ranging from 0 (“I never do this”) to 3 (“I always do this”). In the current study, coping responses about a specific situation were evaluated, with the response options adapted to “I did this” (e.g. 0 “I have never done this”; 3 “I have always done this”). The Portuguese version’s factorial structure replicates the original scale’s characteristics, confirming both the allocation of items to their respective subscales and the internal consistency of its dimensions (Ribeiro & Rodrigues, 2004). Consistent with prior research, the current study demonstrated a good internal consistency, with a Cronbach’s Alpha value of .85 for the total scale. Additionally, as observed by Dias et al. (2012), the scale dimensions were grouped into three categories: Coping focused on the problem (α = .81), Emotion-focused coping (α = .71) and avoidant coping (α = .75) (Dias et al., 2012; Poulus et al., 2020).

***Posttraumatic growthInventory*** (PTGI; Tedeschi & Calhoun, 1996) accesses individuals’ subjective perceptions of positive psychological changes resulting from traumatic life experiences. This 21-item self-report questionnaire employs a Likert scale, ranging from 0 (“I have not experienced this change as a result of the adverse event”) to 5 (“I fully experienced this change as a result of the adverse event”), where higher scores signify greater levels of PTG. In comparison, the original scale comprises five factors: relating to others, personal strength, new possibilities, spiritual change, and appreciation of life. The Portuguese version, as determined through factor analysis, consolidated into four factors: “Perception of personal resources and skills”, “New possibilities and appreciation of life”, “Strengthening interpersonal relationships”, and “Spiritual development”. Demonstrates commendable internal consistency, with a Cronbach’s alpha of 0.96 for the general population (Da Silva et al., 2009). In the present investigation, a comparable level of internal consistency was observed, which remained notably high, with a Cronbach’s alpha value of .95 for the total scale and its constituent dimensions.

### Procedure

The ethical committee of ISPA – Instituto Universitário (Ref.: I-098-12-22) approved the study. All participants signed their informed consent before completing the survey, which was anonymous and voluntary. The survey was implemented with Google Forms, and the order of the instruments was as follows: sociodemographic and grief questionnaire (e.g., age, education, marital status, who is the lost person, how long past over the lost, etc.), the informed consent form and a battery of assessments (IES-R, BriefCOPE, PTGI). The study was disseminated via social networks using paid and specific groups unrelated to grief (e.g., grief and neighbour groups).

### Analysis

The data was analysed with SPSS Statistics 29 software (IBM Corp, Armonk, NY., USA). The first step was to test the metric qualities of the instruments used in this study. To test the model’s validity, a confirmatory factor analysis was carried out using AMOS Graphics 29 software (IBM Corp, Armonk, NY., USA). The procedure followed a “model generation” logic (Jöreskog & Sörbom, 1993). Five fits were merged following the published recommendations (Hu & Bentler, 1999), considering in the analysis of their adjustment the results obtained for the chi-square (χ^2^/df) ≤ 5; for the Tucker Lewis index (TLI) > 0.90; for the comparative fit index (CFI) > 0.90; for the root mean square error of approximation (RMSEA) ≤ 0.08; for the Root Mean Square Residual (RMSR), a smaller value corresponds to a better adjustment.

We also tested whether sociodemographic variables and grief significantly affected PTG using the One-Way ANOVA parametric test. The mediating effects were tested using Path Analysis in the AMOS Graphics 29 software.

## Results

The first step was testing the model’s validity by conducting a 5-factor confirmatory factor analysis. The fit indices obtained are adequate or very close to adequate (χ^2^/gl = 3.28; CFI = 0.87; TLI = 0.87; RMSEA = 0.051; SRMR = 0.124). However, items 13, 18, 20 and 28 had to be removed from the CFE because they had a low factor weight.

### Different Levels of PTG in Sociodemographic and Grief Variables

In the relationship between the age of the sample and PTG (See Table 1), it was found that there is not a significant correlation between age and PTG score and our dimensions. PTGI, *r* (887) = 0,69, p .687. Age was not a statistically significant factor in PTG levels. However, gender was a significant factor in PTGI levels. To study this relationship, 889 participants were recruited (170 men and 719 women). The homogeneity of variances was evaluated using the *Levene* test (*p* > .05). This criterion was violated in Dimensions 1 and 2. Therefore, *t student* test with Welch correction was used in this case. The *t test* showed that women have a higher average in PTGI than men, in PTGI score *t* (887) = −5.45 *p* = .000. It’s important to highlight that the sample used has a big difference in gender, which might affect how reliable these conclusions are. Even though it’s an important and reasonable finding, we need to consider this issue.

**Table 1.** Means Between Groups of Degree of Kinship Variable and Causes of Death in PTG.

Dependent variable	*F*	*p*	Group	Mean	SD
Post traumatic growth	9.64	<0.001	Child	3.56	0.76
			Spouse	3.30	1.06
			Parent	2.61	1.20
			Grandparent	2.39	1.32
			Other	2.49	1.22
	1.16	0.329	Acute illness	2.56	1.24
			Prolonged chronic illness	2.57	1.24
			Accidents	2.93	1.25
			Death by human action	2.71	1.29
			Other	2.51	1.27

A *One-way* ANOVA was used to analyse the relationship between the degree of kinship to the lost person and PTG, and 888 participants were recruited. This variable was found to have a significant effect on PTG (F (4, 884) = 9.64; *p* < .001). When a child or a spouse dies, posttraumatic growthlevels are higher (See Table 1). On the other hand, the cause/reason for which loved ones died did not prove to be a statistically significant factor in PTG levels. The difference between different groups was not significant (F (5, 883) = 1,16; *p* = 0.329) (See Table 1).

### Mediating Effect of Coping in the Relationship with Grief and PTG

The mediating effect of coping on the relationship between IES and PTG was tested through the procedures of Baron and Kenny (1986). The assumptions for carrying out mediation are presented as Supplemental Materials. All the conditions were verified (see complementary materials), except the relationship between IES and PTG mediated by coping strategies, which was not verified in all the variables under study due to some flaws in the assumptions, namely in the variable degree of kinship. When a spouse (Z = 1.77; β = 0.29; *R*^2^ = 0.08; *p =* .076) or a child (Z = 1.13; β = −0.20; *R*^2^ = 0.04; *p =* .258) dies the first assumption failed (direct relationship between IES and PTG) and when the person who dies is in the “other” category the second assumption failed: dimension FPC (Z = 1.92; β = 0.14; *R*^2^ = 0.03; *p =* .054), and EFC (Z = 1.45; β = 0.11; *R*^2^ = 0.01; *p =* .147) (See Table 2).

**Table 2.** Mediating Effects Between of all Variables Under Study and Degree of Kinship.

	Independent Variable	Dependent Variable	z	β	*p*
Parent	IES	PFC	5.11***	0.25	<0.001
	IES	EFC	6.21***	0.30	<0.001
	IES	PTG	2.53*	0.15	0.011
	PFC	PTG	9.83***	0.43	<0.001
	EFC	PTG	4.87***	0.22	<0.001
Grandparent	IES	PFC	6.52***	0.38	<0.001
	IES	EFC	6.29***	0.37	<0.001
	IES	EC	15.21***	0.69	<0.001
	IES	PTG	3.84***	0.28	<0.001
	PFC	PTG	8.49***	0.44	<0.001
	EFC	PTG	2.82**	0.15	0.005
	EC	PTG	−0.45	−0.03	0.665

The Results (See Table 2) suggest that only in the loss of a parent or a grandparent does the mediating effect of *coping.* When the deceased is one of the parents, the IES significantly affects the PFC, the EFC and the dependent variable. The mediating variables PFC and EFC have a significant effect on the dependent variable, so there is a partial mediation effect of PFC and EFC on the relationship between IES and PTG (β1 = 0.29; β2 = 0.15). The addition of *R*^2^ is statistically significant (Δ *R*^2^ = 0.23, *p* < .001), and the model explains PTG variability by 31%.

**Figure 1.**
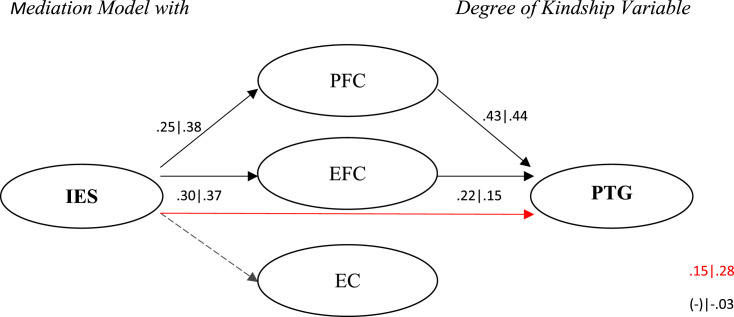
Mediation Model with Degree of Kindship Variable. *Note.* Coefficients on the left relate to the loss of a parent | coefficients on the right relate to the loss of a grandparent.

When a grandparent dies (See Table 2), the IES has a significant effect on the mediating variables and the dependent variable. The mediating variables PFC and EFC have a significant effect on the dependent variable, so there is a partial mediation effect of PFC and EFC on the relationship between IES and PTG (β1 = 0.46; β2 = 0.28). The addition of *R*^2^ is statistically significant (Δ *R*^2^ = 0.20, *p* < .001), and the model explains PTG variability by 41%. (Figure 1).

When the reason for death is an acute illness (See Table 3), the IES significantly affects PFC, EFC and the dependent variable. The mediating variables PFC and EFC have a significant effect on the dependent variable, so there is a partial mediation effect of PFC and EFC on the relationship between IES and PTG (β1 = 0.33; β2 = 0.20). The addition of *R*^2^ is statistically significant (Δ *R*^2^ = 0.21, *p* < .001), and the model explains PTG variability by 32%.

**Table 3.** Mediating Effects Between of all Variables Under Study and Cause of Death.

	Independent Variable	Dependent Variable	z	β	*p*
Acute illness	IES	PFC	5.14***	0.31	<0.001
	IES	EFC	4.72***	0.29	<0.001
	IES	PTG	2.01*	0.15	0.044
	PFC	PTG	6.21***	0.34	<0.001
	EFC	PTG	5.83***	0.32	<0.001
Prolonged chronic illness	IES	FCP	5.55***	0.26	<0.001
	IES	EFC	5.79***	0.27	<0.001
	IES	EC	19.31***	0.68	<0.001
	IES	PTG	4.97***	0.28	<0.001
	PFC	PTG	10.15***	0.41	<0.001
	EFC	PTG	3.98***	0.17	<0.001
	EC	PTG	−0.85	−0.05	0.397
Accidents	IES	PFC	2.03*	0.27	0.042
	IES	EC	4.16***	0.51	<0.001
	IES	PTG	2.00*	0.21	0.033
	PFC	PTG	7.23***	0.65	0.099
	EC	PTG	0.85	0.09	0.396
Others	IES	PFC	3.52***	0.31	<0.001
	IES	EFC	4.45***	0.38	<0.001
	IES	PTG	0.17	0.02	0.865
	PFC	PTG	5.07***	0.40	<0.001
	EFC	PTG	4.17***	0.34	<0.001

When the reason for death is a Prolonged Chronic Illness (See Table 3), the IES has a significant effect on the mediating variables and the dependent variable. The mediating variables PFC and EFC have a significant effect on the dependent variable, so there is a partial mediation effect of PFC and EFC on the relationship between IES and PTG (β1 = 0.38; β2 = 0.28). The addition of *R*^2^ is statistically significant (Δ *R*^2^ = 0.23, *p* < .001), and the model explains PTG variability by 34%.

When the reason for death is an accident (See Table 3), the IES significantly affects PFC, EC and the dependent variable. Only the PFC mediator variable has a significant effect on the dependent variable, so there is a partial mediation effect of PFC on the relationship between IES and PTG (β1 = 0.44; β2 = 0.21). The addition of *R*^2^ is statistically significant (Δ *R*^2^ = 0.42, *p* < .001), and the model explains PTG variability of 62%.

Finally, when the reason for death is “others” (See Table 3), the IES significantly affects PFC, EFC and the dependent variable. The mediation variables PFC and EFC significantly affect the dependent variable, so there is a total mediation effect on the PFC on the relationship between the IES and the PTG (β1 = 0.25; β2 = 0.02). The addition of *R*^2^ is statistically significant (Δ *R*^2^ = 0.25, *p* < .001), and the model explains PTG variability of 31%.

## Discussion

The present study investigated posttraumatic growth (PTG) in bereaved individuals and explored the pathways leading to this growth. The findings indicated that, following grief, most individuals experienced positive changes, like PTG. While previous research has hinted at potential factors mediating PTG (Michael & Cooper, 2013), these findings remain debatable.

In terms of gender, it was observed that women exhibited higher levels of PTG than men. Women engage in significantly more deliberate rumination (Treynor et al., 2003). This type of rumination plays a crucial role in the reconstruction and restoration of a viable belief system following an event such as the death of a loved one and, therefore, a greater PTG experience (Vishnevsky et al., 2010). Additionally, women are more inclined to utilise emotion-focused cognitive strategies (Thoits, 1991; Vingerhoets & Van Heck, 1990), promoting improved emotional management and the maintenance of emotional equilibrium. Given that PTG arises from active engagement with the consequences of a traumatic event, women tend to engage in deeper contemplation of the event and cognitive elaboration, resulting in a heightened experience of PTG. It is important to highlight that our sample exhibits gender disparity (i.e., with a higher proportion of women), urging caution when interpreting these results. However, they should be considered as they align with previous research. In any case, future research with representative samples should be conducted to confirm the present results.

In the context of the degree of kinship with the deceased, a distinct association was highlighted between certain types of loss and the phenomenon of PTG, particularly in the case of the loss of a child or a spouse. These findings appear to deviate from prior research, which has indicated that the death of a child is typically followed by heightened levels of suffering and depression (Devries & Arbuckle, 2005; Stroebe & Schut, 2001b). Additionally, the loss of a spouse has traditionally been linked to a decreased likelihood of positive transformations due to the complexity of grief involving multiple concurrent losses (Leahy, 1997). Nonetheless, this discovery aligns with the fundamental principles of the theory for this phenomenon. According to Tedeschi and Calhoun (1995), individual beliefs is a key factor in determining PTG. Considering this perspective, when an event of this nature challenges and disrupts one’s core beliefs about the world and their place within it, the individual not only grapples with the immediate distress associated with the event but also engages in a profound process of reevaluating and reconstructing their guiding principles. While the death of a child or a spouse may indeed bring about deeper suffering and heightened challenges, it paradoxically appears to stimulate a greater inclination toward PTG, as suggested by Tedeschi and Calhoun (2006).

When examining the cause of death, no statistically significant correlations were found, which opens the door to several possible interpretations for this outcome. Ultimately, although there is widespread agreement on the notion that violent causes of death, such as suicide, homicide, and fatal accidents, are associated with unfavourable grief outcomes (Sanders, 1988; Stroebe & Schut, 2001a), these findings were not confirmed in the present study. These results may be understood considering that each type of death has unique characteristics influencing coping. Additionally, although the sample of this study included cases of violent deaths resulting from human actions or accidents, they were a minority when compared to deaths categorised as more “natural”.

PTG is a multidimensional construct involving cognitive restructuring processes (Tedeschi & Calhoun, 1996, 2004a, 2006). In this context, the second objective aimed to comprehend the mediating role of coping strategies between the impact of loss and PTG. It was observed that greater impact is associated with higher levels of growth. While this may be unexpected, these results are consistent with previous findings, emphasising the importance of disruption in an individual’s beliefs in post-trauma reconstruction. Loss severity has been found to correspond to higher growth levels (Armstrong & Shakespeare-Finch, 2011). In other words, the greater the perceived threat and damage, the stronger the association with growth (Znoj, 2005).

In analysing the mediating role of coping while considering the degree of kinship with the deceased person, it was observed that when such a relationship exists, it is partial and only at the level of problem-focused coping (PFC) and emotional-focused coping (EFC), as opposed to the EC (avoidant coping). Although seemingly incongruent, it has been found that in the loss of a parent or grandparent, coping plays a mediating role between the impact of the loss and PTG, but the levels of growth are lower. Conversely, in the death of a spouse or child, coping does not prove significant for the experience of PTG, but the growth is higher. Several reasons may explain this phenomenon, such as the intensity of the loss. This intensity, though variable, implies a disruption of fundamental beliefs and pre-established meanings, leading to an inevitable need for reconstruction, which subsequently gives rise to PTG (Kállay, 2007; Linley & Joseph, 2002). Therefore, losing a spouse or child is possibly a significant rupture in prior beliefs due to it being less normative than, for example, the death of a parent or grandparent. Therefore, it requires an inevitable and immediate reconstruction, relegating coping strategies to a secondary role. This allows us to hypothesise that different losses with respect to kindship constitute different degrees of challenge. Therefore, individuals can mobilise adaptive coping strategies and restore their previous levels, even if their growth is less pronounced.

Concerning the cause of death, it was found that regardless of the type of death, the PFC is the most significant in this mediation, followed by the EFC. The impact of the loss continues to be associated with higher levels of post-traumatic growth, as we can see in other studies where supportive coping and active coping were each associated with higher PTG, and avoidant coping was associated with lower PTG (Fisher et al., 2020). However, when the cause of death is acute illness or prolonged chronic disease, the relationship is less intense due to the impact of coping strategies. In other words, these types of deaths seem to require a greater effort in using more adaptive coping strategies to face the loss. However, given the irregularity and uniqueness of the grieving process, the results appear to require both problem-focused and emotion-focused strategies, which may co-occur throughout the process. Therefore, the individual differences that can be observed in the adaptive potential must be understood, considering the interactive and procedural nature, the importance attributed to the problem and the capacity or lack of control that the individual has over that same problem (Folkman & Moskowitz, 2004; Lazarus, 1993b).

Regarding accidental deaths, a partial relationship was observed between the impact of the loss and the PTG and only at the PFC level. Events that occur unexpectedly tend to be considered more traumatic and, in the early stages, give rise to both a heightened perception of threat and significant levels of despair that can influence the experience of PTG (Linley & Joseph, 2004). However, this threat ultimately generates an almost innate need to mobilise some strategy to cope with such an event. This is where active problem-solving comes into play, involving positive cognitive restructuring and problem resolution, which enhances individuals’ ability to engage in new opportunities and, ultimately, PTG (Wolchik et al., 2008).

In conclusion, regardless of the degree of kinship and the cause of death, PFC and EFC prove to be essential for the experience of PTG in the grieving process, in contrast to avoidant coping, which aligns with previous research (S Lipp & O’Brien, 2022). These findings may be justified by the fact that problem-focused coping requires an active role in managing problem resolution (Billings & Moos, 1981; Lazarus & Folkman, 1984), and emotion-focused coping involves regulating the emotions resulting from the same event (Billings & Moos, 1981). Therefore, these two types of strategies, which tend to be more adaptative, require the processing of the event, which consequently enhances PTG. In contrast, avoidant coping, as it involves suppressing or avoiding the emotions resulting from the event (Suar et al., 2017), hinders the processing of the event and prevents the experience of PTG.

This research had several limitations. Firstly, the convenience nature of the sample raises the potential for bias. For example, it has a higher proportion of female participants, which may influence the observed relationships. Replicating this study with an equal representation of both men and women would be valuable. The replication of this study involving a comparative analysis between a community sample and a clinical cohort is also important. Finally, this is a crossectional study, which limits the causal interpretations of these associations. Longitudinal studies may provide further insight into the nature of the observed relationships. Besides these limitations, it is important to highlight that some of the methodological choices are relevant in drawing conclusions for this study. A broad sample with respect to the type or time since death is important in studying coping and PTG in an unrestricted way. Future research studying sub-groups may find important variations. The same is true for kindship. Losing a parent in one’s twenties or fifties may be substantially different. Finer studies or with wider samples may expand the present exploratory study.

## Conclusion

There are different trajectories following a loss, and the discovery that individuals who experienced greater suffering also experienced higher levels of posttraumatic growth allows us to infer that both processes are compatible rather than antagonistic and can, in the end, foster growth. This study suggests that cognitive processing and subsequent coping strategies used by individuals in the grieving process are crucial pathways to PTG.

## Supplemental Material

Supplemental Material - From Grief to Growth: The Role of Coping Strategies, Kinship and Cause of Death Supplemental Material for From Grief to Growth: The Role of Coping Strategies, Kinship and Cause of Death by Marta Pereira, Ana Moreira, and David Dias Netoa in OMEGA - Journal of Death and Dying.

## Data Availability

The datasets used and/or analyzed during the current study are available from the corresponding author on reasonable request.*
