# Supplementary material for: From Grief to Growth: The Role of Coping Strategies, Kinship and Cause of Death
Source: Omega (Westport). 2024 Jun 6;93(2):1233–49. doi: 10.1177/00302228241259647 (PMC13176474; doi:10.1177/00302228241259647)
Supplement: Supplemental Material - From Grief to Growth: The Role of Coping Strategies, Kinship and Cause of Death [file sj-pdf-1-ome-10.1177_00302228241259647.pdf]

Supplementary materials

Table 1

Associations between IES and all variables

Group		Independent variable	Dependent variable	Z	β	R2	p
Reason od death	Acute Illness	IES	PFC	5.14***	0.31	0.10	< 0.001
			EFC	4.72***	0.29	0.08	< 0.001
			EC	13.97***	0.66	0.44	< 0.001
			PTG	5.43***	0.33	0.11	< 0.001
	Prolonged Chronic Illness	IES	PFC	5.56***	0.26	0.07	< 0.001
			EFC	5.80***	0.27	0.07	< 0.001
			EC	19.37***	0.68	0.46	< 0.001
			PTG	8.54***	0.38	0.14	< 0.001
	Accidents	IES	PFC	2.04*	0.27	0.08	0.042
			EFC	2.21*	0.30	0.09	0.027
			EC	4.17***	0.51	0.25	< 0.001
			PTG	3.53***	0.44	0.20	< 0.001
	Death by human action	IES	PFC	3.13**	0.52	0.27	0.002
			EFC	3.96***	0.61	0.37	< 0.001
			EC	4.85***	0.68	0.47	< 0.001
			PTG	3.52***	0.56	0.32	< 0.001
	Others	IES	PFC	3.53***	0.31	0.09	< 0.001
			EFC	4.46***	0.38	0.14	< 0.001
			EC	10.06***	0.67	0.45	< 0.001
			PTG	2.79**	0.25	0.06	0.005
Degree of relatedness	Parents	IES	PFC	5.11***	0.25	0.06	< 0.001
			EFC	6.21***	0.30	0.09	< 0.001
			EC	18.02***	0.68	0.46	< 0.001
			PTG	5.88***	0.29	0.08	< 0.001
	Grandparents	IES	PFC	6.52***	0.38	0.14	< 0.001
			EFC	6.29***	0.37	0.13	< 0.001
			EC	15.22***	0.69	0.47	< 0.001
			PTG	8.21***	0.46	0.21	< 0.001
	Spouse	IES	PFC	-0.28	-0.05	0.00	0.777
			EFC	1.49	0.25	0.06	0.137
			EC	4.49***	0.61	0.37	< 0.001
			PTG	1.77	0.29	0.08	0.076
	Child	IES	PFC	1.32	0.24	0.06	0.186

			EFC	1.20	0.22	0.05	0.229
			EC	3.67***	0.56	0.31	< 0.001
			PTG	-1.13	-0.20	0.04	0.258
	Other	IES	PFC	1.92	0.14	0.03	0.054
			EFC	1.45	0.11	0.01	0.147
			EC	10.23***	0.61	0.37	< 0.001
			PTG	3.41***	0.25	0.06	< 0.001

The results indicate that, given any of the reasons for death, the IES has a significant and positive association with all dimensions of coping and with post-traumatic growth.

With regard to the degree of relatedness, when the person who died is a parent or a grandparent, there is also a positive and significant association between the IES, all dimensions of coping and the PTG. When the person who died is a spouse or a child, the IES only has a positive and significant effect on EC. When the person who died is in the "other" category, IES has a positive and significant effect on the EC and PTG.

Finally, both for the situation of expected/unexpected death and the both of genders, the IES has a significant and positive association with all dimensions of coping and PTG.

Table 2

Associations between Coping and and PTG

Group		Independent Variable	Dependent Variable	Z	R2	β	p
Reason of death	Acute Illness	PFC	PTG	4.78***	0.39	0.34	< 0.001
		EFC		4.47***		0.31	< 0.001
		EC		1.71		0.09	0.087
	Prolonged Chronic Illness	PFC	PTG	8.05***	0.35	0.42	< 0.001
		EFC		3.40***		0.19	< 0.001
		EC		3.26**		0.13	0.001
	Accidents	PFC	PTG	4.89***	0.62	0.64	< 0.001
		EFC		0.95		0.12	0.344
		EC		2.03*		0.18	0.043

	Death by human action	PFC	PTG	1.91	0.52	0.40	0.056
		EFC		1.39		0.27	0.166
		EC		1.13		0.17	0.261
	Other	PFC	PTG	3.53***	0.41	0.37	< 0.001
		EFC		3.08**		0.32	0.002
		EC		0.10		0.01	0.918
Degree of relatedness	Parents	PFC	PTG	7.52***	0.36	0.41	< 0.001
		EFC		3.98***		0.22	< 0.001
		EC		1.41		0.06	0.159
	Grandparents	PFC	PTG	6.37***	0.42	0.46	< 0.001
		EFC		2.37*		0.17	0.018
		EC		2.83**		0.14	0.005
	Spouse	PFC	PTG	0.83	0.42	0.16	0.409
		EFC		2.84**		0.54	0.005
		EC		-0.40		-0.06	0.686
	Child	PFC	PTG	1.86	0.20	0.42	0.063
		EFC		0.17		0.04	0.864
		EC		-1.01		-0.17	0.310
	Other	PFC	PTG	3.62***	0.35	0.28	< 0.001
		EFC		4.45***		0.34	< 0.001
		EC		2.83**		0.17	0.005

When the reason of death is an acute illness or “other” cause both PFC and EFC have a positive and significant association with PTG. When it comes to a Prolonged Chronic Illness, all the dimensions of coping have a positive and significant association with PTG. When the reason of death is an accident, only PFC and EC have a positive and significant association with PTG.

With regard to the degree of relatedness, when one of the parents dies, both PFC and EFC have an positive and significant association in the PTG. When the death is of a grandparent or “other”, all the dimensions have a significant and positive association with PTG. Finally, when a spouse dies, only EFC has a significant and positive association with PTG.
